# 4A syndrome: ocular surface investigation in an Italian young patient

**DOI:** 10.1186/1471-2415-14-155

**Published:** 2014-12-08

**Authors:** Pasquale Aragona, Laura Rania, Anna Maria Roszkowska, Domenico Puzzolo, Antonio Micali, Antonina Pisani, Giuseppina Salzano, Maria Francesca Messina

**Affiliations:** Department of Experimental Medical-Surgical Sciences, Ocular Surface Diseases Unit, University of Messina, Via Consolare Valeria 1, I-98125 Messina, Italy; Department of Biomedical Sciences and Morphofunctional Imaging, University of Messina, Via Consolare Valeria 1, I-98125 Messina, Italy; Department of Pediatric, Gynecological, Microbiological and Biomedical Sciences, University of Messina, Via Consolare Valeria 1, I-98125 Messina, Italy

**Keywords:** Allgrove’s 4A syndrome, *In vivo* corneal confocal microscopy, Conjunctival impression cytology, Dry eye, Tear function

## Abstract

**Background:**

Allgrove’s 4A syndrome determines ocular surface changes. This is the first report providing an up-to-dated analysis of the ocular surface in an affected patient.

**Case presentation:**

An 18-years-old male Caucasian patient, with a complex progressive gait disorder and adrenal insufficiency, was referred for ophthalmic evaluation, as part of the clinical assessment. He underwent the following tests: best corrected visual acuity, tear osmolarity, tear film break-up time (BUT), corneal fluorescein staining, Schirmer’s I test, lid margin assessment, corneal sensitivity, *in vivo* corneal confocal microscopy, conjunctival impression cytology, tonometry and fundus exam. A dry eye condition was documented by the Schirmer’s I test of 0 mm/5’ in both eyes, accompanied by tear hyperosmolarity, mild meibomian gland dysfunction, reduced BUT, mucus filaments in the tear film and conjunctival epithelium metaplasic changes. The corneal confocal microscopy showed the presence of activated keratocytes, while the nerve pattern was normal.

**Conclusions:**

The dry eye in this patient appears to be due to tear aqueous deficiency and can be considered as part of the 4A syndrome. The decreased tear production, resulting from a deterioration of the autonomic innervation of the lacrimal glands rather than an impaired corneal innervation, can be considered as part of the systemic autonomic dysfunction present in this disease.

## Background

Allgrove syndrome, or triple A, is a clinical entity characterized by the association of deficient cortisol secretion, with elevated plasma corticotrophin, achalasia of the cardia and defective or absent tear production [1]. A disturbance of ATP/cyclic AMP metabolism of the adrenal glands, leading to autonomic symptoms, was proposed. Allgrove syndrome was entered in the Online Mendelian Inheritance in Man compendium with the number #231550. The association of the triple A syndrome with autonomic and peripheral neuropathies was termed as 4A syndrome [2].

Even if alacrima is the most constant symptom (75% of the patients) [3], thus indicating an important ocular surface involvement, previous ophthalmological investigations were limited to the fundus exam, Schirmer’s I test and slit lamp examination of the ocular surface. The fundus exam evidenced a bilateral nerve head pallor [4], the Schirmer’s I test showed reduced or minimal [5] tear secretion, and a punctate keratopathy was often observed [6]. However, no data are available, as far as we know, about the exact ocular surface features, which may guide appropriate diagnosis and treatment in a clinical setting [7].

We report the ocular surface features including also tear osmolarity, *in vivo* corneal confocal microscopy and conjunctival impression cytology, to obtain clinical information useful to demonstrate and follow up ocular surface alterations in a patient with 4A syndrome.

## Case presentation

The patient, an 18 years old Caucasian boy, is the second child of consanguineous parents (second degree cousins). His medical history was unremarkable until the age of 7 years, when he developed progressive gait, running disturbances and difficulties in playing soccer. At the age of 9.7 years, he experienced a severe ketotic, hypoglycemic attack. At the age of 10 years, due to the progressive worsening of the neurological symptoms, he was referred to the Unit of Neurology of the University of Messina, where a complex picture of upper and lower motor neuron involvement was recognized. Magnetic resonance imaging of the brain and cervical spinal cord was normal. On that occasion, the presence of marked skin pigmentation and brown spots of the tongue, together with a clinical history positive for ketotic hypoglycemia, suggested the diagnosis of adrenal insufficiency. This clinical suspicion was confirmed by a typical hormonal profile with very high ACTH levels and undetectable levels of cortisol, both basal and stimulated by i.v. ACTH. Further investigations showed an autonomic dysfunction with postural hypotension and abnormal heart reflexes. The oral treatment with hydrocortisone, at substitutive doses (10 mg/m^2^/day), caused a significant improvement of the patient’s general well being, although ACTH levels remained elevated. The coexistence of ACTH-resistant adrenal insufficiency and neurological manifestations suggested the hypothesis of triple A syndrome, that was confirmed by DNA analysis of the *AAAS* gene. In fact, sequencing the coding regions and exon/intron junctions of this gene identified a homozygous splice mutation in intron 14. This is a G > A replacement at the first nucleotide in intron 14, resulting in aberrant splicing (IVS14 + 1G > A). As expected, the parents were both heterozygous carriers of the mutation, as well as the two clinically healthy siblings [8].

During the clinical assessment, an ophthalmologic exam was performed, because the patient referred of symptoms characterized by foreign body sensation and itching, starting in the morning and worsening in the evening, mild dryness and light sensitivity. Before the clinical exam, an informed consent was obtained. The clinical procedures were in accordance with the Declaration of Helsinki.

The ophthalmologic exam included visual acuity assessment, ocular surface study, tonometry, and fundus examination. For the ocular surface study, the following tests were performed: tear osmolarity measurement (TearLab™ Osmolarity System Inc., TearLab™ Corp., San Diego, CA, USA), slit-lamp examination of the ocular surface, tear film break-up time (BUT), corneal fluorescein staining, Schirmer’s I test (SNO strips, Laboratoire Chauvin, Aubenas, France), lid margin assessment, corneal sensitivity, *in vivo* corneal confocal microscopy (Confoscan 4, Nidek, Vigonza PD, Italy) and conjunctival impression cytology (Supor 200 membrane filters, Gelman Sciences, Ann Arbor, MI, USA) [9, 10].

**Figure 1:**
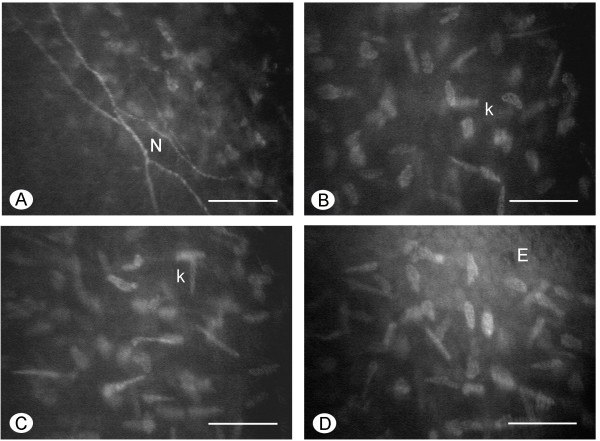
**Confocal microscopy of the cornea. A**. The subbasal nerves (N) present a rectilinear course with some beadings. **B**. In the anterior stroma, keratocytes (k) show morphological features typical of activated cells. **C**. In the posterior stroma, keratocytes (k) possess elongated processes and well evident nuclei. **D**. Endothelial cells (E) are normal. Scale bar: 50 μm.

**Figure 2:**
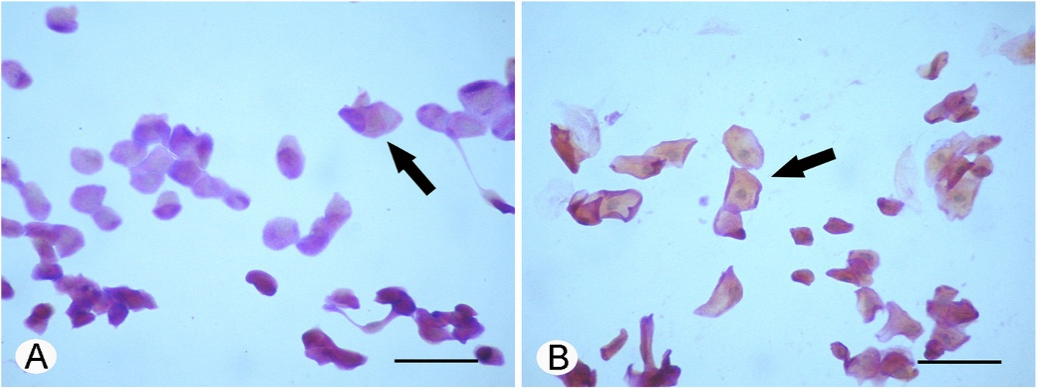
**Conjunctival impression cytology specimens stained with PAS-Papanicolaou stain. A**. In right eye, isolated cells show condensed nuclear chromatin and mild keratinisation (arrow). **B**. In the left eye, isolated cells show a metaplasic appearance, pyknotic nuclei with reduced N/C and highly keratinized cytoplasm (arrow). Scale bar: 40 μm.

The best-corrected visual acuity was 20/20 in both eyes. Tear osmolarity was 300 mOsm/l in the right eye (RE) and 332 mOsm/l in the left eye (LE). The slit-lamp examination showed an unstable tear film with a BUT of 2 sec in both eyes. A mild conjunctival fluorescein staining was present in the LE conjunctiva in both interpalpebral areas; no signs of epithelial damage were present in the RE. A mild papillary hypertrophy was present in the upper tarsal conjunctiva in both eyes. Schirmer’s I test was 0 mm/5’ in both eyes. The lid margins showed a mild hyperemia, with meibomian gland expression giving rise to slightly turbid meibum. The keratoaesthesiometry was within normal values (RE = 1.08 g/mm^2^; LE = 0.96 g/mm^2^). In both eyes, the corneal confocal microscopy showed normal epithelial features. The subbasal corneal nerves presented a rectilinear course, with occasional beadings (Figure 1A). Stromal keratocytes, either in the anterior (Figure 1B) or in the posterior stroma (Figure 1C), showed morphological features typical of activated cells. Endothelial cells were normal (Figure 1D). The conjunctival impression cytology showed in RE the presence of islands of bilayered epithelial cells, together with areas of isolated cells. The cells within the bilayered tissue appeared of normal size, with N/C of 1:1 or 1:2. Also the isolated cells showed a normal N/C, although cells with reduced N/C could be seldom observed. Nuclear chromatin showed a condensed appearance. Goblet cells were normally represented. A mild keratinisation was present in some cells (Figure 2A). The LE showed the presence of islands of bilayered cells, together with areas of isolated and highly keratinized cells with a metaplasic appearance and pyknotic nuclei. Particularly in the isolated cells, the N/C was reduced (Figure 2B). In the multilayered areas some goblet cells were observed. The intraocular pressure was 14 mmHg in both eyes. The fundus exam was normal in both eyes.

## Conclusions

4A syndrome, a rare disease, is characterized by an autonomic disturbance, which is usually demonstrated by peripheral motor and sensory neuropathy, involving several organs. Although one of the more frequent dysfunctions of this syndrome is the alacrima, its pathogenic mechanism is still not clear [3]. In fact, due to the lack of in depth studies, it is not known whether the ocular surface damages derive either from a systemic autonomic dysfunction of the lacrimal gland or from primarily altered ocular surface structures leading to a lacrimal gland impairment. In our patient, we found a markedly decreased tear production, as demonstrated by Schirmer’s I test, which was accompanied by increased tear osmolarity, metaplasic conjunctival changes and corneal keratocytes activation, in presence of normal corneal nerves and aesthesiometry. Therefore, it seems that, in our patient, the ocular surface dysfunction is part of the autonomic disturbance, which may directly determine a lacrimal gland impairment, rather than a primary corneal nerve involvement. These findings suggest that the dry eye present in this patient is typically of the aqueous deficient type. The lacrimal gland alterations described elsewhere [4, 11] might depend on an innervation failure, which is always accompanied by gland atrophy [12]. Therefore, the alacrima demonstrated in Allgrove syndrome could be considered as a part of a systemic autonomic dysfunction, rather than an independent topical clinical feature. It could be proposed that the treatment of the ocular surface dysfunction in these patients should consider, together with topical treatments aimed to protect the corneal-conjunctival epithelium, also a systemic approach addressed to improve the lacrimal gland secretion and correct its neurological dysfunction.

## Consent

Written informed consent was obtained from the patient for publication of this Case report and any accompanying images. A copy of the written consent is available for review by the Editor of this journal.
